# Massive tubercular pseudo-tumor of the thigh: a case report

**Published:** 2012-06-11

**Authors:** Bhavuk Garg, Soumya Chakraborty, Vijay Kumar, Rajesh Malhotra

**Affiliations:** 1 Department of Orthopaedics, All India Institute of Medical Sciences, India; 2 Department of Orthopaedics, Postgraduate Institute of Medical Education and Research, India

**Keywords:** Tuberculosis, pseudo-tumor, thigh

## Abstract

Psoas abscess in cases of tuberculosis originates from the primary lesion in the lower dorsal or the lumbar spine. From the spinal origin of the Psoas muscle, this abscess tracks down its sheath and may be palpable in the iliac fossa, in the lumbar triangle, in the upper part of thigh below the inguinal ligament. We present a rare case, where patient presented with thigh swelling, which on first look gave an impression of a malignant origin. But subsequent investigation revealed it to be one of tuberculous origin, and that to, tracking down of a Psoas abscess. According to best of our knowledge, there has been no reported case of a Psoas abscess tracking down to the thigh and knee and mimicking a tumour.

## Introduction

Ilio-Psoas abscess is an important disease with subtle and often nonspecific presentation that frequently provides a diagnostic challenge. Before the discovery of modern antitubercular treatment, Ilio-Psoas abscess was characteristically a well recognised complication of tuberculosis of the spine. However with the decreasing prevalence of tuberculosis, iliopsoas abscess is becoming uncommon in the developed countries. However with increasing incidence of HIV infection, a recent surge of tuberculosis has been noted, especially with multi-drug resistant *Mycobacterium tuberculosis* [1].

In this communication, we report the first case of tuberculous ilio-psoas abscess, to the best of our knowledge, masquerading as massive tumor of thigh in an immunocompetent host, caused by multi-drug-resistant *Mycobacterium tuberculosis*.

## Patient and observation

**Figure 1:**
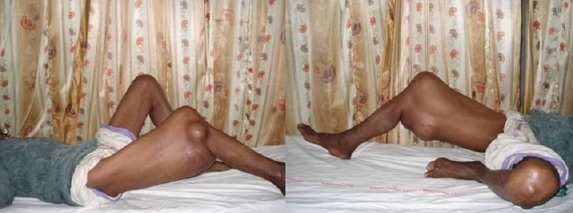
Clinical photograph showing massive swelling of right thigh, mimicking a soft tissue tumor

A 52 year old male patient was referred to our institute as a massive tumor of right thigh (Figure 1) for further management. There was no history of fever or loss of appetite; however history for night sweats and weight loss and low back pain was positive. He denied any history of cough, shortness of breath, hemoptysis or history of contact with a tuberculous patient or relative. There were no other medical illnesses.

Physical examination revealed normal vital signs. The right thigh was massively swollen and was non tender and was not associated with any inflammation. The patient looked ill and underweight, but there was no lymph node enlargement or clubbing. Chest and cardiovascular examination was unremarkable. Abdominal examination revealed soft, non-tender abdomen and no organomegaly. Palpation of lumbar spine revealed tenderness (Figure 1).

**Figure 2:**
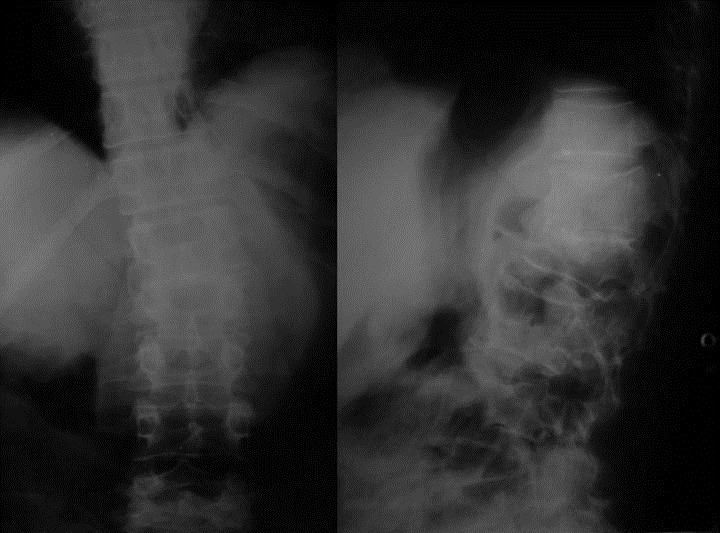
Anteroposterior and lateral radiograph of lumbar spine of same patient showing Pott's spine involving L1 and L2

**Figure 3:**
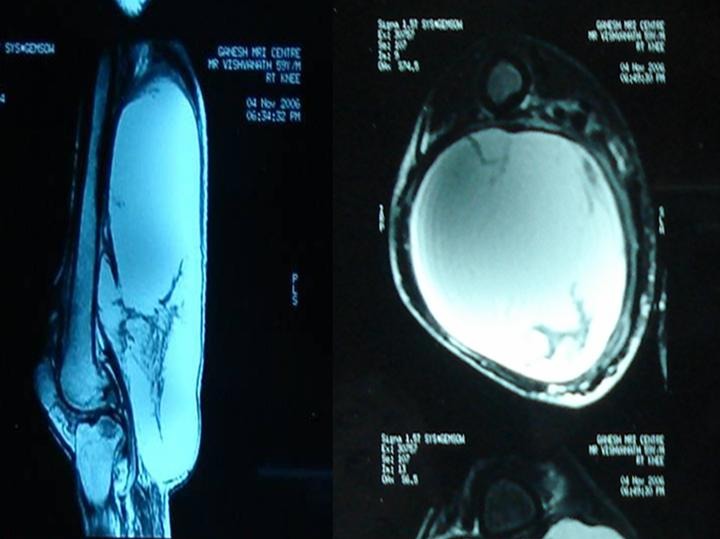
Sagittal and transverse section of right thigh showing large abscess on magnetic resonance imaging (MRI)

Laboratory investigations revealed normal full blood count, liver enzymes, electrolytes, urea and creatinine. ESR was 85 and HIV serology was negative. X-rays of the chest, pelvis and thigh was normal. X-rays of the lumbar spine (Figure 2) revealed decreased disc space between L1 and L2 vertebra along with collapse of L2. Magnetic resonance imaging of thigh (Figure 3) revealed large well encapsulated abscess in the back of thigh extending upto popliteal fossa, in continuation with ilio-psoas abscess (Figure 2 and Figure 3).

The abscess was aspirated under ultrasound guidance. Ziehl- Neelsen stain of the aspirate was positive for acid-fast bacilli. Culture of the aspirate revealed *Mycobacterium tuberculosis* resistant to isoniazid, and rifampicin, but sensitive to ethambutol, pyrazinamide, streptomycin, capreomycin, ofloxacin and amikacin. He was started on pyrazinamide, ethambutol, streptomycin, ofloxacin and amikacin. Isoniazid and rifampicin were discontinued. The abscess was then drained under ultrasound guidance and 650 ml of purulent fluid was obtained. Streptomycin was locally instilled and the drain was kept in place for seven days. A period of 18 months of therapy was completed. At the end of the treatment course, the swelling resolved completely, the patient improved clinically, gained weight and was walking normally.

## Discussion

The clinical presentation of iliopsoas abscess is often variable and non-specific [2]. The classical clinical triad consisting of fever, back pain, and limp is present in only 30% of the patients with iliopsoas abscess [3]. Most commonly, it presents in atypical ways. A diligent physical examination is essential for the prompt diagnosis of this condition. Patients may present with a painless swelling below the inguinal ligament. This may be confused with a femoral hernia or enlarged inguinal lymph nodes [2]. In this instance, it presented as a massive swelling involving the whole thigh, mimicking a soft tissue tumor. A large iliopsoas abscess may present with deep venous thrombosis. The cause of the thrombosis is due to extrinsic compression of the iliac vein from the iliopsoas abscess [4, 5].

Along with usual laboratory and routine radiographs, Magnetic resonance imaging should be done for definitive diagnosis because of better discrimination of soft tissues and the ability to visualize the abscess wall and the surrounding structures without the need of a intravenous contrast medium [6]. Ultrasound is inexpensive, has no radiation effects, and is easy to perform but is extremely operator dependent and is diagnostic in only 60% of the cases [7]. It is also a useful adjunct in drainage of abscess.

Treatment involves the use of appropriate antitubercular drugs along with drainage of the abscess. Depending on the results of the abscess fluid culture and sensitivity, adjustments should be made. Drainage of the abscess can be done under ultrasound or computed tomography guidance. Tuberculosis is considered as medical disease and is usually well controlled by antitubercular drugs. Indications for surgery [2] for ilio-psoas abscess are limited and include (a) failure of percutaneous drainage, (b) relative contraindication of percutaneous drainage, for example, clotting disorders, and (c) the presence of an another intra-abdominal pathology which requires surgery.

## Conclusion

This is the first case of its kind, where ilio-psoas abscess presented as massive swelling of the thigh, mimicking as a soft tissue tumor. It is prudent that this condition is recognised and managed promptly. Early management and drainage of the abscess reduces the morbidity and mortality.
